# The Use of Social Media in Interprofessional Education: Systematic Review

**DOI:** 10.2196/11328

**Published:** 2019-01-11

**Authors:** Audra Rankin, Maria Truskey, Margaret S Chisolm

**Affiliations:** 1 School of Nursing Johns Hopkins University Baltimore, MD United States; 2 Welch Medical Library Johns Hopkins University School of Medicine Johns Hopkins University Baltimore, MD United States; 3 Psychiatry and Behavioral Sciences Johns Hopkins University School of Medicine Johns Hopkins University Baltimore, MD United States

**Keywords:** interprofessional education, interprofessional learning, medicine, nursing, social media, social networking

## Abstract

**Background:**

The implementation of interprofessional education (IPE) activities into health care education is a challenge for many training programs owing to time and location constraints of both faculty and learners. The integration of social media into these IPE activities may provide a solution to these problems.

**Objective:**

This review of the published literature aims to identify health care IPE activities using social media.

**Methods:**

The authors searched 5 databases (from the beginning coverage date to May 27, 2017) using keywords related to IPE and social media. Teams of 2 authors independently reviewed the search results to identify peer-reviewed, English language papers reporting on IPE activities using social media. They assessed the study quality of identified papers using the Medical Education Research Study Quality Instrument.

**Results:**

A total of 8 studies met the review’s inclusion criteria. Of these 8 papers, 3 had single-group, posttest-only study design; 4 had single-group, pre- and posttest design; and 1 had nonrandomized 3-group design. Qualitative and quantitative outcome measures showed mixed results with the majority of student feedback being positive.

**Conclusions:**

Despite a need for additional research, this review suggests that the use of social media may aid the implementation of health care IPE.

## Introduction

The need to integrate interprofessional education (IPE) activities into health care education is a topic of growing importance. Members of the Interprofessional Education Collaborative, including national associations such as the American Association of Colleges of Nursing and Association of American Medical Colleges, value IPE and encourage institutions to create opportunities for health care trainees and practitioners from different disciplines to learn with, from, and about each other [1]. Although it is important to train health care professionals to work together effectively, many training programs have difficulty implementing IPE activities owing to schedule and location conflicts, lack of faculty support, and financial constraints [2].

The use of social media may provide a solution to many of the challenges inherent to IPE encountered by health care educators. Commonly used for personal social networking, social media tools provide a unique way for IPE learners to broaden their professional networks without the time and location constraints inherent to in-person networking [3]. In addition, these tools encourage a workforce prepared to adopt—with ease—new technologies for communication and care coordination [4]. Health care educators need to know the best ways to use social media in IPE to implement rigorous multidisciplinary learning. To address this gap in the literature, this review aims to identify papers describing studies of the use of social media in health care IPE and outcomes of social media use in IPE.

## Methods

We searched the literature in 5 key databases (PubMed, Cumulative Index to Nursing & Allied Health Literature, Education Source, Education Full Text, and Academic Source Complete) from each database’s beginning coverage date through our search date (May 27, 2017) for papers on the use of social media in health care IPE. The databases were selected because of their comprehensive representation of available peer-reviewed journals, as well as their dedicated scope in the relevant disciplines to this topic (eg, medicine, nursing, allied health, and education). Key search concepts were selected with their Medical Subject Headings terms and other controlled vocabulary equivalents, as well as related keywords—“social media” OR “social networking” AND “interprofessional education (IPE)” OR “interprofessional learning” (Multimedia Appendix 1).

We then transferred titles and abstracts identified by this search into a reference management system. Two authors (ANR and MSC) independently evaluated each abstract to determine whether the paper potentially met the following *a priori*-determined inclusion criteria: accessible full-text, English language, peer-reviewed journal, IPE learners, social media educational intervention evaluation, and focus on IPE. If either reviewer found the abstract to meet the inclusion criteria, the paper advanced to full-text review and its PDF version retrieved and attached within the reference manager. Two authors (ANR and MSC) then independently evaluated the PDF of each full-text paper for inclusion. If the paper did not meet the inclusion criteria, each reviewer specified the reason for exclusion. A third reviewer (MT) resolved discordant reviewer responses.

Two authors (ANR and MSC) independently extracted relevant data from the included paper. The original version of the Kirkpatrick Model was used to assess each study’s outcome measures. Reviewers identified each study’s Kirkpatrick hierarchy level [5] and, on the Web-based software platform Covidence.org, assessed the quality of each study using the validated Medical Education Research Quality Instrument (MERSQI) for Quantitative Studies [6]. Discordant reviewer responses for data extraction, Kirkpatrick hierarchy level, and MERSQI scores were resolved by consensus of the authors. Kirkpatrick’s hierarchy was used to identify learning outcomes ranging from learner participation to changes in patient outcomes. In addition, Kirkpatrick’s Learning Model evaluates training programs’ impact on a hierarchy of 4 levels—perceptions (level 1) to outcomes (level 4). MERSQI scores were based on the study design, sampling, type of data, validity of an evaluation instrument, data analysis, and outcomes; scores could range from 5 to 18.

## Results

**Figure 1:**
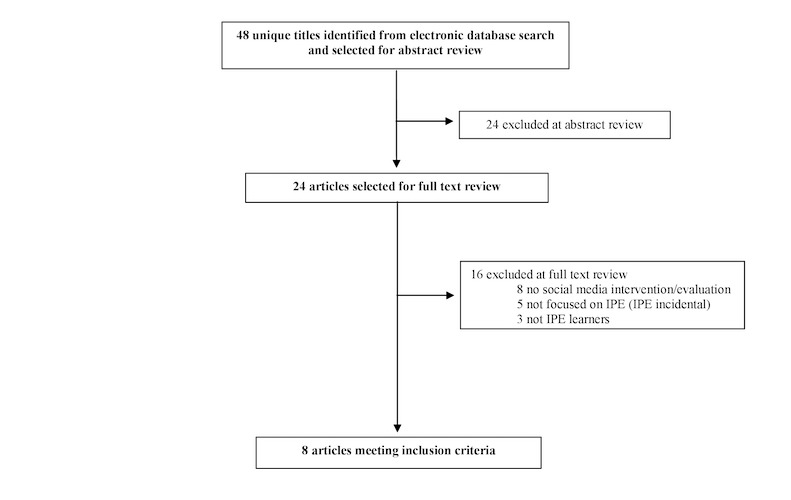
The flow diagram of search methods and results of the review on social media use in interprofessional education.

Our initial database search identified 48 unique titles, of which we selected 24 for full-text review (Figure 1). After full-text review, we determined that 8 papers met our inclusion criteria [2-4,6-11]. Of 8 studies identified by this review, 4 were single-group, posttest-only in design [3,4,10,11], 3 were single-group, pre- and posttest [7,8,9], and 1 was a nonrandomized 3-group study [2]. Multimedia Appendix 2 summarizes the results.

Study participants encompassed students from a broad range of professions as follows: nursing (7 studies), medicine (5 studies), pharmacy (5 studies), occupational therapy (2 studies), dentistry (2 studies), dental hygiene (1 study), physical therapy (1 study), radiography (1 study), and public health (1 study). A variety of social media tools and software were used as part of the educational intervention, with some studies using multiple tools as follows: blogs (2 studies), Wikis (2 studies), discussion boards (2 studies), Ning (1 study), and Second Life (1 study). Studies used quantitative (1 study), qualitative (4 studies), and mixed-method (3 studies) outcome measures. Most measured outcomes at the level of learner perceptions (5 studies), a few at the level of learner social media use behavior (3 studies), and none at the level of skills or patient outcomes. The included studies MERSQI scores ranged from 6 to 11.5.

The one single-group, posttest-only study that reported quantitative results found that 68% of students deposited information on (ie, contributed to) the Wiki, 42% edited the Wiki, and 20% visited the Wiki only to view content [10]. All remaining single-group, posttest studies reported qualitative results in the form of learner reflections. One study reported that students found the intervention to include respectful, engaging interaction among team members [3]. One study reported student reflections on learning, improvement, and innovations. These covered a range of topics, including culture, impact on practice, and module delivery [11], all of which received mixed student reviews. Furthermore, one study reported that the majority of student reviews were positive [4].

Of 3 single-group, pre- and posttest studies, only one compared learners’ pre- and posttest on validated quantitative measures [Interdisciplinary Education Perception Scale (IEPS) and Readiness for Interprofessional Learning Scale (RIPLS)] [9]. We found no significant difference before or after the intervention (*P*>.10). Of the other 2 single-group, pre- and poststudies, one compared medical, nursing, and pharmacy students on quantitative social media use metrics and found significant differences in the duration of use in weeks 1, 2, and 4 between medicine and both nursing and pharmacy students (*P*<.05) [7]. In addition, 2 of the 3 single-group, pre- and posttest studies reported qualitative results [8,9]. One had all students respond to a postintervention survey instrument and found that they were fairly split on whether the intervention should be used again, although many students had positive remarks on the flexibility the intervention provided and the impact on their learning [9]. The other reported extremely positive feedback with all participants wanting to continue paired learning in their professional development [8].

Only one study, which received the highest MERSQI score, was designed to compare multiple interventions delivered to interprofessional groups of learners and found no significant differences in IEPS and RIPLS scores among the 3 groups, although no *P* value was reported [2]. In addition, these 3 groups did not significantly differ in their social media use metrics (the number of posts and time spent on site) showed (*P*=.47).

## Discussion

### Principal Findings

Many interprofessional learners are familiar with the personal use of social media, particularly the use of social networking sites. One study found that 90% of students used social media regularly, and many students report they engage in social networking despite a heavy academic load [9]. Social media tools, such as Facebook, Instagram, and Twitter, are often used for personal use with users developing informal personal networks [7]. Transitioning learners from personal to professional use of social media to establish interprofessional learning communities provides a unique path forward for IPE [7].

The included studies used a variety of methods to integrate social media into IPE activities and, thus, bring together learners from a wide range of health care disciplines. Paired learning, virtual simulation platforms, blogs, and Wikis were among the social media tools used. Health care educators developed IPE activities with the intention of increasing learner knowledge and clinical skills in an interprofessional environment. Competencies from the Accreditation Council for Pharmacy Education, American Association of Colleges of Nursing, and the Interprofessional Education Collaborative were considered in the development of learning activities [4,9]. The evaluation included qualitative and quantitative feedback, such as the IEPS and the RIPLS survey instruments [2], as well as pre- and postexperience survey instruments.

Feedback from learners was often positive, with many stating social networking activities provided a unique opportunity for collaboration, allowing them to gain unique perspectives from other disciplines in a flexible format [4,9,10]. Although most learners were more than willing to collaborate, some felt that the use of social networking platforms had a “laid-back” and less academic feel [9]. Creating social media activities embedded in core courses, containing practical or clinical purpose, and with adequate pedagogical supports are recommended [2].

### Limitations

Although this review identified only a limited number of studies, this most likely reflects the emerging nature of the use of social media in IPE. More rigorous research on the effectiveness of social media tools, as well as future Web-based tools in IPE, needs to be conducted. A thorough discussion of this review in the context of IPE literature cannot be performed, as there are currently no published reviews on this topic. However, systematic reviews on social media use in medical and nursing education are available. The use of social media in these fields has been associated with improved knowledge, attitudes, and skills, as well as the promotion of learner engagement and professional development [11,12]. Although studies used a variety of social media tools, evidence as to which of these is the most effective in an IPE activity at improving learner behaviors and positively impacting patient outcomes is still lacking. Another limitation of this review is that the most updated version of the Kirkpatrick model (the New World Kirkpatrick Model) was not used, which may have affected the results.

Many studies in the review evaluated participant feedback rather than formally assessing the acquired knowledge. Although the papers mention the need to consider a variety of professional competencies in curriculum planning, limited information was available on how those learner competencies were evaluated. In addition, evaluation criteria were often informal and provided limited information on learning outcomes. The lack of consistency between methods used to evaluate learner outcomes is an inherent limitation of the review. Additional feedback, including how social media can be used to transform learning environments into a space that flattens professional hierarchical structure, would be valuable [2]. Moreover, information on how to engage and support faculty to create social learning experiences that have academic rigor would be helpful [13]. Although social media in IPE has been used to overcome time and space limitations, a gap in the literature exists on measurable improvement in overcoming these challenges.

### Conclusions

This review provides valuable information on the variety of social media tools available and presents a good case for the use of social media to overcome many challenges with IPE learning activities, including schedules, meeting locations, and limited faculty and financial support. Overall, learner feedback was positive with many studies highlighting the flexibility of the learning environment. Although additional evidence is needed, these findings suggest that the integration of social media into interprofessional learning activities can be a valuable health care teaching method.

## Appendix

Multimedia Appendix 1 Search terms for review on social media use in interprofessional education (IPE).

Multimedia Appendix 2 Major findings and quality assessment of studies of social media use in interprofessional education identified by the review, grouped by the study design.

## Abbreviations

IEPS: Interdisciplinary Education Perception Scale
IPE: Interprofessional education
MERSQI: Medical Education Research Quality Instrument
RIPLS: Readiness for Interprofessional Learning Scale
