# Supplementary material for: The Use of Social Media in Interprofessional Education: Systematic Review
Source: JMIR Med Educ. 2019 Jan 11;5(1):e11328. doi: 10.2196/11328 (PMC6329891; doi:10.2196/11328)
Supplement: Multimedia Appendix 1 [file mededu_v5i1e11328_app1.pdf]

**Multimedia Appendix 1.** Search terms for review on social media use in interprofessional education (IPE).

PubMed

(“IPE”[tiab] OR ((“interprofessional”[tiab] OR “inter-professional”[tiab] OR “inter professional”[tiab]) AND (communication*[tiab] OR relation*[tiab] OR educat*[tiab] OR train*[tiab] OR program*[tiab] OR workshop*[tiab] OR seminar*[tiab] OR learn*[tiab]))) AND (“Social Media”[MeSH] OR “social media”[tiab] OR “social medium”[tiab] OR “social mediums”[tiab] OR “social networking”[tiab] OR “twitter”[tiab] OR “Web 2.0”[tiab] OR “facebook”[tiab] OR wiki*[tiab] OR blog*[tiab])

CINAHL

(DE “Interprofessional education” OR AB (“IPE”) OR TI (“IPE”) OR AB ((“interprofessional” OR “inter-professional” OR “inter professional”) AND (communication* OR relation* OR educat* OR train* OR program* OR workshop* OR seminar* OR learn*)) OR TI ((“interprofessional” OR “inter-professional” OR “inter professional”) AND (communication* OR relation* OR educat* OR train* OR program* OR workshop* OR seminar* OR learn*))) AND (DE “Social media” OR DE “Backchannels (Social media)” OR DE “Online chat” OR DE “Social media in education” OR DE “Wikis (Computer science)” OR AB (“social media” OR “social medium*” OR “social networking” OR “twitter” OR “facebook” OR “blog*” OR “wiki*”) OR TI (“social media” OR “social medium*” OR “social networking” OR “twitter” OR “facebook” OR “blog*” OR “wiki*”))

Education Source

(DE “Interprofessional education” OR AB (“IPE”) OR TI (“IPE”) OR AB ((“interprofessional” OR “inter-professional” OR “inter professional”) AND (communication* OR relation* OR educat* OR train* OR program* OR workshop* OR seminar* OR learn*)) OR TI ((“interprofessional” OR “inter-professional” OR “inter professional”) AND (communication* OR relation* OR educat* OR train* OR program* OR workshop* OR seminar* OR learn*))) AND (DE “Social media” OR DE “Backchannels (Social media)” OR DE “Online chat” OR DE “Social media in education” OR DE “Wikis (Computer science)” OR AB (“social media” OR “social medium*” OR “social networking” OR “twitter” OR “facebook” OR “blog*” OR “wiki*”) OR TI (“social media” OR “social medium*” OR “social networking” OR “twitter” OR “facebook” OR “blog*” OR “wiki*”))

Education Full Text

(DE “Interprofessional education” OR AB (“IPE”) OR TI (“IPE”) OR AB ((“interprofessional” OR “inter-professional” OR “inter professional”) AND (communication* OR relation* OR educat* OR train* OR program* OR workshop* OR seminar* OR learn*)) OR TI ((“interprofessional” OR “inter-professional” OR “inter professional”) AND (communication* OR relation* OR educat* OR train* OR program* OR workshop* OR seminar* OR learn*))) AND (DE “Social media” OR DE “Backchannels (Social media)” OR DE “Online chat” OR DE “Social media in education” OR DE “Wikis (Computer science)” OR AB (“social media” OR “social medium*” OR “social networking” OR “twitter” OR “facebook” OR “blog*” OR “wiki*”) OR TI (“social media” OR “social medium*” OR “social networking” OR “twitter” OR “facebook” OR “blog*” OR “wiki*”))

Academic Source Complete

(DE “SOCIAL media” OR DE “BACKCHANNELS (Social media)” OR DE “BLOGS” OR DE “COMPUTER bulletin boards” OR DE “FALSE news (Social media)” OR DE “ONLINE chat” OR DE “ONLINE social networks” OR DE “PODCASTS” OR DE “SOCIAL bookmarks” OR DE “SOCIAL media in education” OR DE “SOCIAL media in medicine” OR DE “THEATER & social media” OR DE “WIKIS (Computer science)” OR AB (“social media” OR “social medium*” OR “social networking” OR “twitter” OR “facebook” OR “blog*” OR “wiki*”) OR TI (“social media” OR “social medium*” OR “social networking” OR “twitter” OR “facebook” OR “blog*” OR “wiki*”)) AND (DE “Interprofessional education” OR AB (“IPE”) OR TI (“IPE”) OR AB ((“interprofessional” OR “inter-professional” OR “inter professional”) AND (communication* OR relation* OR educat* OR train* OR program* OR workshop* OR seminar* OR learn*)) OR TI ((“interprofessional” OR “inter-professional” OR “inter professional”) AND (communication* OR relation* OR educat* OR train* OR program* OR workshop* OR seminar* OR learn*)))
