# Supplementary material for: The Use of Social Media in Interprofessional Education: Systematic Review
Source: JMIR Med Educ. 2019 Jan 11;5(1):e11328. doi: 10.2196/11328 (PMC6329891; doi:10.2196/11328)
Supplement: Multimedia Appendix 2 [file mededu_v5i1e11328_app2.pdf]

Multimedia Appendix 2. Major findings and quality assessment of studies of social media use in interprofessional education identified by the review, grouped by the study design.

First Author, Date	Participant number and type	Social media tools	Outcome measures	Outcomes	Kirkpatrick hierarchy level	MERSQI score
Single-group, posttest only						
King, 2009	620 total from all disciplines, including nutrition, medicine, dentistry, dental hygiene, nursing, pharmacy, physical therapy, occupational therapy, and medical laboratory science; 357 medicine, nursing, and pharmacy students (for a subset of evaluation outcomes)	Social networking site	Site usage metrics such as site areas accessed, duration of site visit (minutes/Student)	Most accessed site areas: curriculum; duration of use in weeks 1, 2, and 4: significant differences between medicine and both nursing/pharmacy (P<.05); F_{2502}=7.63 for week 1; F_{2502}=4.19 for week 3; F_{2502}=6.18 for week 4	Behaviors	8
Davidson, 2011	100 medicine, 84 nursing, 23 occupational therapy students	Discussion board	Postexperience reflections	All students reported respectful, engaging interaction	Perception s	7
Evans, 2014	60 medicine, nursing, paramedic, pharmacy, and physiotherapy students	Blog, Wiki, and discussion board	Postexperience reflections on learning, improvement, and innovations including culture/barriers, impact on practice, and module delivery	Mixed student reviews in all areas	Perception s	6.5
Skiba, 2014	Graduate nursing and pharmacy	Cerner AES and Second	Postexperience reflections	Majority of students were positive about	Perception s	6

	students, Project 1 (*N*=23), Project 2 (*N*=8)	Life		intervention		
Single-group, pre- and posttest						
Pittenger, 2013	17 nursing and 34 pharmacy students	Web-based collaborativ e tools	IEPS and RIPLS; postexperience survey	IEPS and RIPLS: no significant differences (*P*>.1); qualitative postexperience survey: 42% students endorsed that the strategy should be used again but 58% disagreed	Perception s	11.5
Stephens, 2013	150 nursing and radiography students	Wiki	Postexperience survey; views/visits, edits, and other usage metrics	Usage metrics: 68% reported depositing information; 42%, editing; 20%, viewing only	Behaviors	7.5
Kelly, 2014	60 “junior doctors” and nonclinical managers	Blog	Likert scale based on NHS Leadership competencies	Improvement in perceived preparedness in all competency areas	Perception s	6.5
3-group nonrandomized						
Pittenger, 2013	18 (16 completed) medicine, nursing, dentistry, pharmacy, veterinary medicine, and public health students. Three groups were comprised of 6 students each. One student in	Ning social networking platform	IEPS and RIPLS; the number of posts and time spent; postexperience survey	IEPS and RIPLS: no significant difference, no *P* value reported; the number of posts and time spent: no significant difference (*P*=.467); postexperience survey: all students in the highly structured group indicated a positive impact on knowledge,	Behaviors	11

	each group represented medicine, nursing, dentistry, pharmacy, veterinary medicine, and public health.			students in the minimally structured group did not indicate a positive impact on knowledge		
